# Supplementary material for: Structural and functional analyses of hepatitis B virus X protein BH3-like domain and Bcl-xL interaction
Source: Nat Commun. 2019 Jul 19;10:3192. doi: 10.1038/s41467-019-11173-1 (PMC6642116; doi:10.1038/s41467-019-11173-1)
Supplement: Supplementary file 1 — Supplementary Information [file 41467_2019_11173_MOESM1_ESM.pdf]

Supplementary information

Structural and functional analyses of the HBx BH3-like domain and its interaction with Bcl-xL

Tian-Ying Zhang[1,2,†], Hong-Ying Chen[3,†], Jia-Li Cao[1,2,†], Hua-Long Xiong[1,2], Xiao-Bing Mo[3], Tian-Liang Li[4], Xiao-Zhen Kang[1,2], Jing-Hua Zhao[1,2], Bo Yin[3], Xiang Zhao[5], Cheng-Hao Huang[1,2], Quan Yuan[1,2,*], Ding Xue[4,5,*], Ning-Shao Xia[1,2,*], Y. Adam Yuan[3,6]

[1] State Key Laboratory of Molecular Vaccinology and Molecular Diagnostics, School of Public Health & School of Life Sciences, Xiamen University, Xiamen 361102, China

[2] National Institute of Diagnostics and Vaccine Development in Infectious Diseases, School of Public Health & School of Life Sciences, Xiamen University, Xiamen 361102, China

[3] National University of Singapore (Suzhou) Research Institute, 377 Lin Quan Street, Suzhou Industrial Park, Jiangsu, 215123, China

[4] Department of Molecular, Cellular and Developmental Biology, University of Colorado, Boulder, Colorado 80309, USA

[5] School of Life Sciences and Collaborative Innovation Center for Diagnosis and Treatment of Infectious Diseases, Tsinghua University, Beijing 100084, China.

[6] Department of Biological Sciences and Centre for Bioimaging Sciences, National University of Singapore, 14 Science Drive 4, Singapore, 117543, Singapore

[†] T.Y.Z., H.Y.C. and J.L.C contributed equally to this work.

[*] To whom correspondence should be addressed. E-mail: yuanquan@xmu.edu.cn, ding.xue@colorado.edu, or nsxia@xmu.edu.cn.

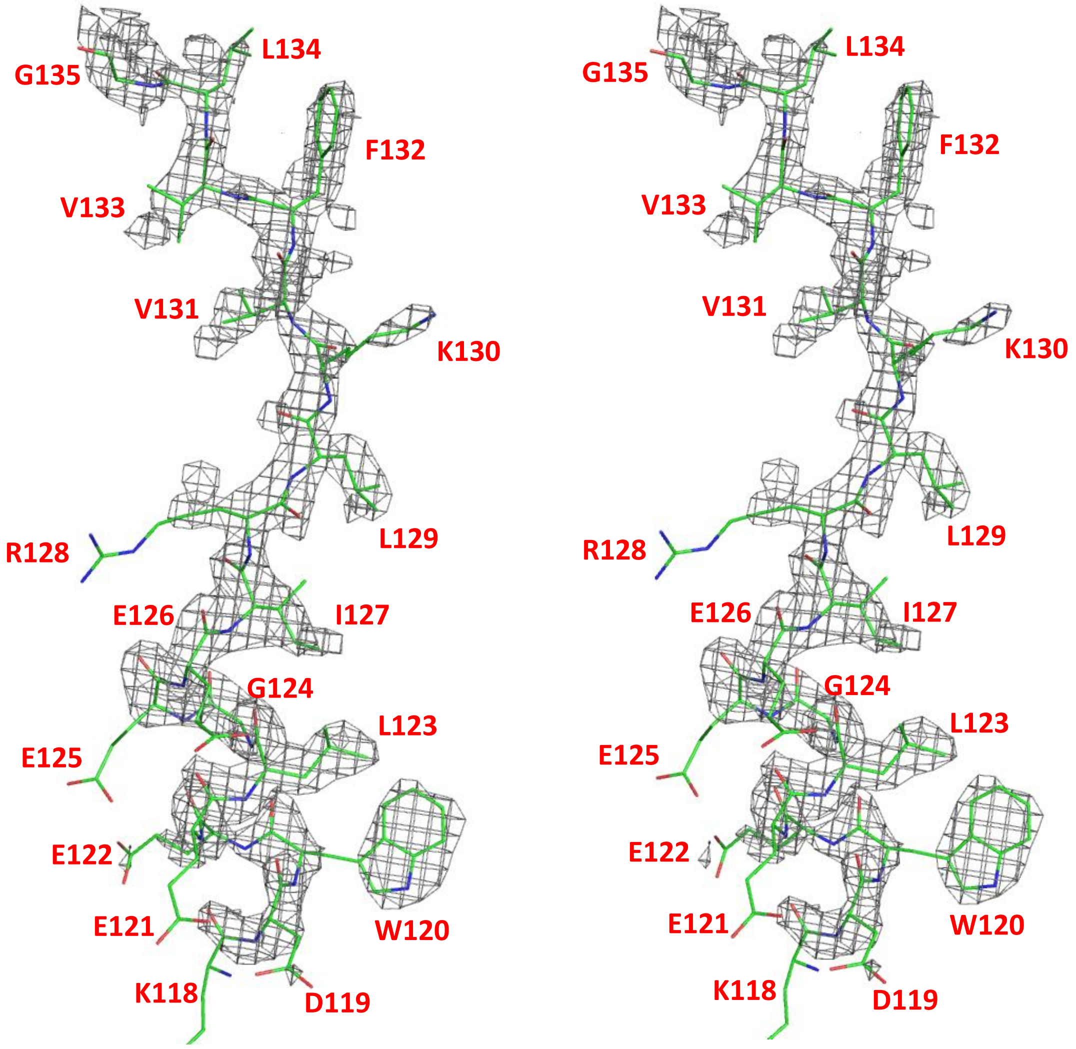

G135
L134
F132
V133
V131
K130
R128
L129
E126
I127
G124
L123
E125
E122
W120
E121
K118
D119

Supplementary Figure 1. Stereoview of HBx 113-135 peptide structure within the Bcl-xL/HBx model

The models of HBx-aa113-135 peptides (residues 118-135 are traceable and built) are colored in green and the density maps are colored in grey. The simulated annealing omit maps were calculated using Fo-Fc coefficients and phases derived from 40 rounds of simulated annealing calculation of the model without HBx. The map is contoured at 2σ.

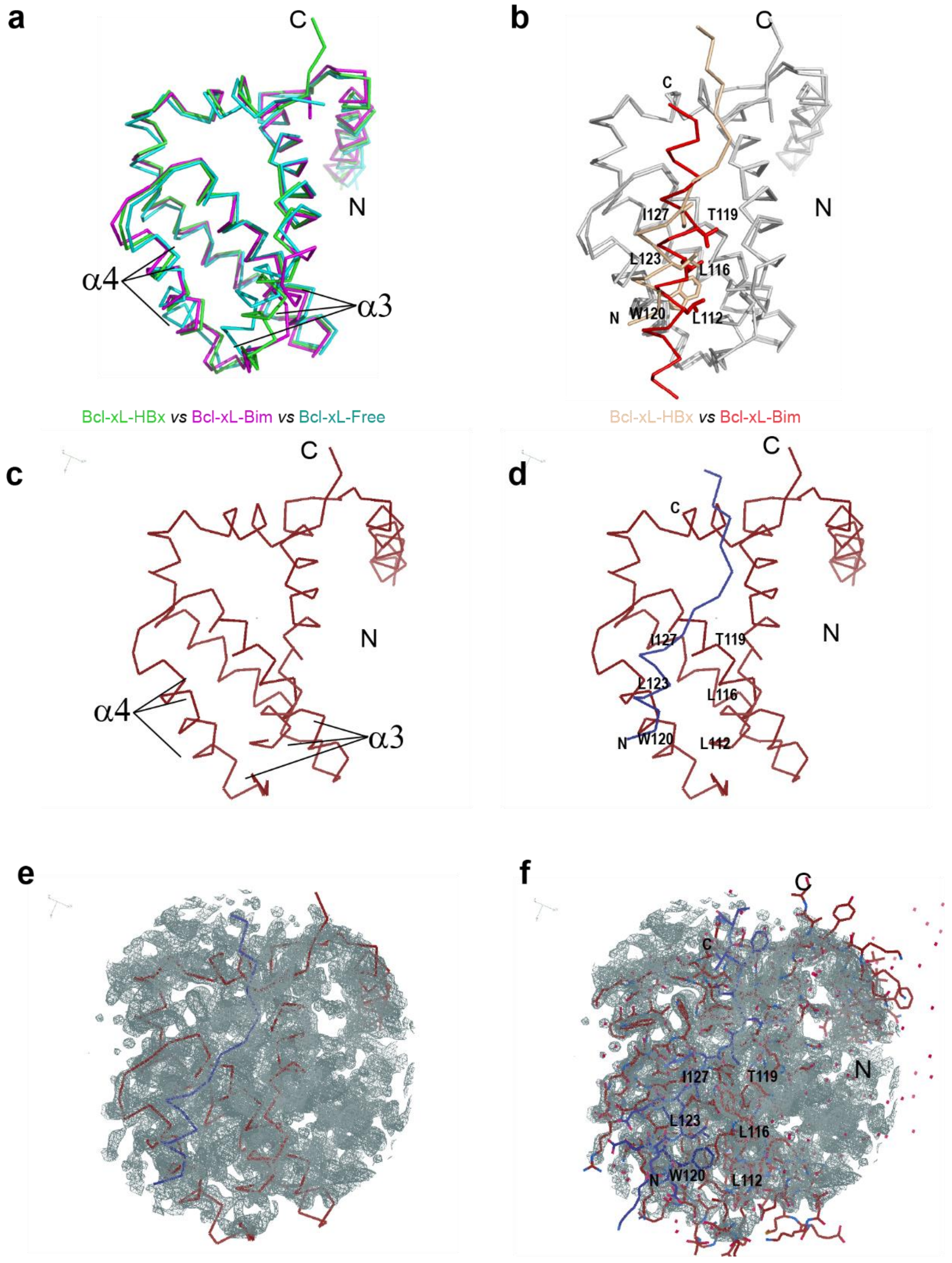

Supplementary Figure 2. Structural superimpositions of the Bcl-xL/HBx-BH3 motif complex, the Bcl-xL/Bim-BH3 motif complex, and Bcl-xL at free form. a Ribbon representation of the Bcl-xL structures. The structures of the Bcl-xL/HBx-BH3 motif complex (PDB ID 5B1Z), the Bcl-xL/Bim-BH3 motif complex (PDB ID 1PQ1[10]),

and Bcl-xL at free form (PDB ID 1R2D[49]) are colored in green, magenta and cyan, respectively. The bound BH3 peptides are omitted from the figure for clarification. Significant structural movements are observed at the α 3 and α 4 regions in the Bcl-xL/HBx-BH3 motif complex. **b** Structural comparison of the HBx-BH3 and Bim-BH3 peptides upon Bcl-xL binding. The bound HBx-BH3 motif is colored in wheat and the bound Bim-BH3 motif is colored in red, whereas Bcl-xL is colored in grey. Compared to the location of the Bim-BH3 peptide, the HBx-BH3 peptide shifts ~5Å towards α 4 and ~2Å towards the termini. Hence, the HBx-BH3 helix interacts with Bcl-xL using different sets of side chains. **c** Cartoon representation of crystal structure of Bcl-xL. The c-terminal, N-terminal, α3 and α4 are labeled. **d** Cartoon representation of crystal structure of Bcl-xL and HBx peptide complex. The c-terminal, N-terminal, α3 and α4 are labeled. The peptide chains of HBx are indicated. Bcl-xL and HBx peptide are labeled in brown and blue, respectively. **e** The electron densities around the Bcl-xL/HBx in cartoon mode are shown in gray mesh, with contour level = 0.48 e/Å3 (1.49 rmsd). **f** The electron densities around the Bcl-xL/HBx residue are shown in gray mesh, with contour level = 0.48 e/Å3 (1.49 rmsd).

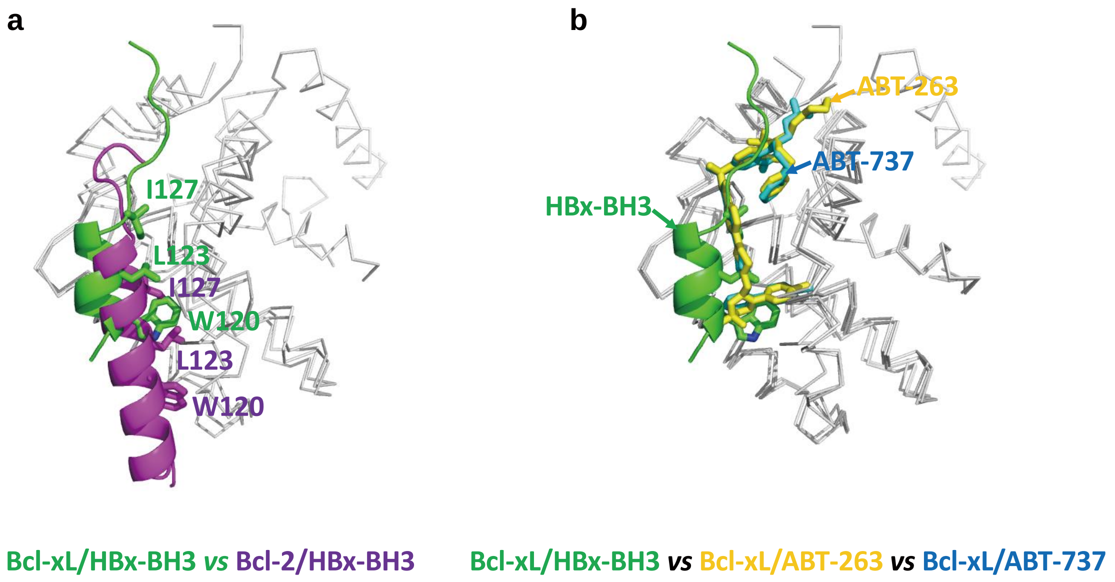

Supplementary Figure 3. Structural superimpositions of complex structures of Bcl-xL/HBx-aa113-135, Bcl-xL/ABT-263, Bcl-xL/ABT-737 and Bcl-2/HBx-aa110-135. a Structural superposition of Bcl-xL/HBx-BH3 (PDB ID 5B1Z) and Bcl-2/HBx-BH3 (PDB ID 5FCG[9]). Structures of Bcl-xL and Bcl-2 are shown in ribbon mode and colored in grey, whereas the HBx BH3-like motifs are shown in cartoon mode and colored in green (in complex with Bcl-xL) and magenta (in complex with Bcl-2), respectively. The side chains of the key residues in BH3-like motifs are indicated in stick mode. Notably, these two HBx BH3-like motifs with virtually the same sequences adopt two significantly different secondary structures. **b** Structural superposition of Bcl-xL/ABT263 (PDB ID 4QNQ), Bcl-xL/ABT737 (PDB ID 2YXJ[21]), and Bcl-xL/HBx BH3-like structures. The Bcl-xL proteins are shown in ribbon mode and colored in grey, whereas the HBx BH3-like motif is shown in cartoon mode and the side chains of the key residues in the HBx BH3-like motif and ABT molecules are indicated in stick mode. Notably, ABT molecules and the HBx BH3-like peptide target different grooves and pockets.

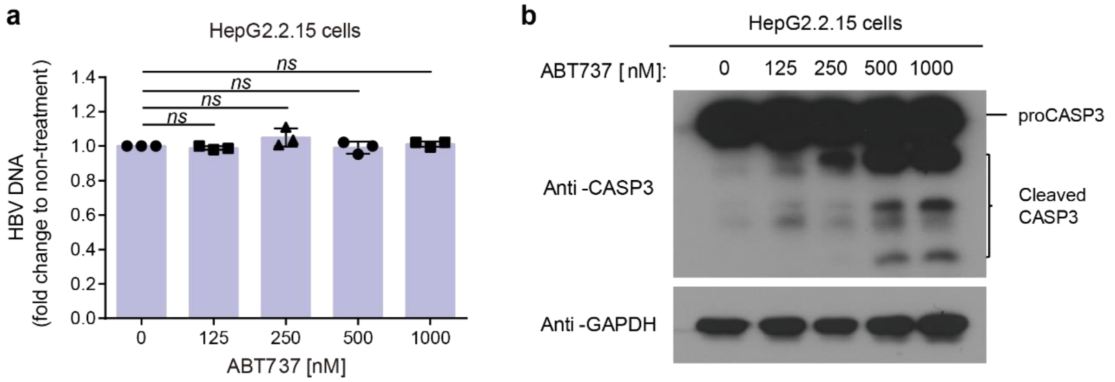

Supplementary Figure 4. ABT-737 do not inhibit HBV replication in HepG2.2.15 cells. a HepG2.2.15 cells were treated with 125 nM, 250 nM, 500 nM and 1000 nM of ABT-263 or ABT-737 for two days. Cytoplasmic HBV viral particles were isolated and the viral DNA replication intermediates were quantified by real-time PCR using a specific primer set targeting HBS ORF (Materials and Methods). The results represent the fold change of the replicative intermediates in HepG2.2.15 cells from the indicated treatments, compared with those without treatment. **b** HepG2.2.15 cells were treated with the indicated concentrations of ABT-263 or ABT-737 for two days. The lysate was analyzed by immunoblotting using antibodies to Caspase-3 (CASP3) and GAPDH. Data shown represent mean ± SD. Significance is indicated on the top. Not significant (ns): $P > 0.05$; two-tailed unpaired *t*-tests. Source data are provided as a Source Data file.

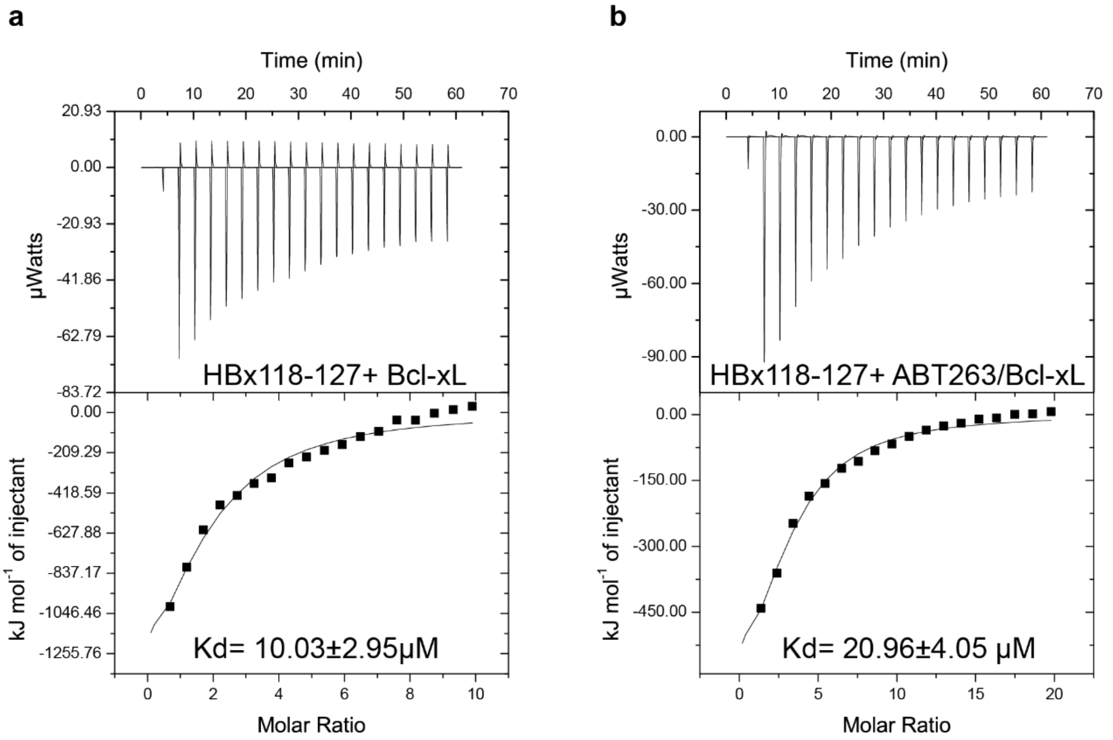

Supplementary Figure 5. ITC data of HBx-aa118-127 binding with Bcl-xL alone and Bcl-xL saturated by ABT263, respectively. Raw titration data and integrated heat measurements are shown in the upper and lower plots, respectively. **a** ITC result shows the dissociation constant between HBx-aa118-127 and Bcl-xL is 10.03±2.95 μM; **b** ITC result shows the dissociation constant between HBx-aa118-127 and Bcl-xL, which was firstly saturated by ABT263, is 20.96±4.05 μM.

a b c

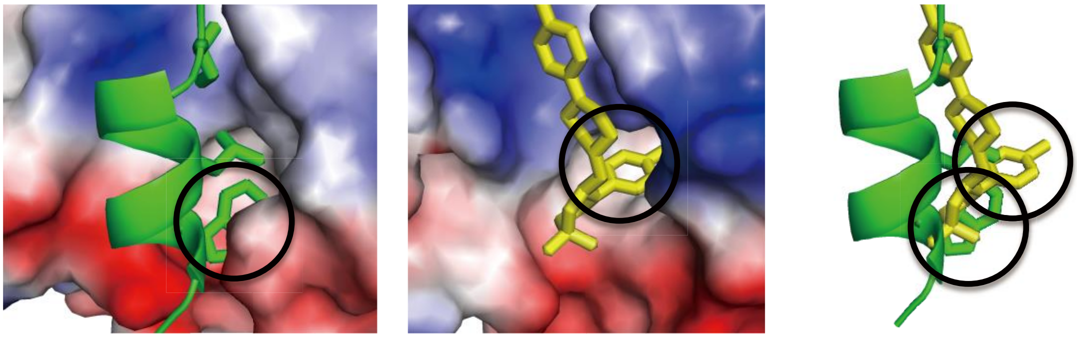

Trp120 side chain binding pocket | ABT263 “chloride-benzene” moiety binding pocket | Structural superimposition

Supplementary Figure 6. Structural comparison of Bcl-xL binding pockets for HBx-aa118-127 and ABT-263. a A close-up view of the Bcl-xL binding pocket for HBx-aa118-127. The structure of Bcl-xL is in surface mode and the HBx-aa118-127 is in cartoon mode. **b** A close-up view of the Bcl-xL binding pocket for ABT-263. The structure of Bcl-xL is in surface mode and the ABT-263 molecule is in stick mode. **c** Structural superimposition of Bcl-xL-bound HBx-aa118-127 and ABT-263. The Bcl-xL binding pockets for HBx-aa118-127 and ABT-263 are indicated by black circles, which are largely nonoverlapping.

Supplementary Table 1 Solvent-Accessible Surface Area Calculation

**5B1Z**	solvent-accessible surface area			Buried surface area
	Chain/Bcl-xL: 10186.49	Chain/HBx-BH3: 2527.419	Chains/Bcl-xL+HBx-BH3: 10699.15	2014.76
**4QNQ**	solvent-accessible surface area			Buried surface area
	Chain/Bcl-xL: 8190.94	Chain/ABT263: 1643.32	Chains/Bcl-xL+ABT263: 8009.24	1825.02
**2YXJ**	solvent-accessible surface area			Buried surface area
	Chain/Bcl-xL: 8059.996	Chain/ABT737: 1202.395	Chains/Bcl-xL+ABT737: 7783.147	1479.25
**5FCG**	solvent-accessible surface area			Buried surface area
	Chain/Bcl2: 9290.396	Chain/HBx-BH3: 3339.15	Chains/Bcl2+HBx-BH3: 9851.63	2777.912

Supplementary Methods

Northern Blot Analysis of HBV RNA. Total RNA was extracted from transfected cells using the TRIzol® Reagent (Invitrogen, #223412) according to the manufacturer's instructions. 20 μg of total RNA was loaded and separated by electrophoresis on a 1% denaturing formaldehyde agarose gel, then transferred onto nylon membranes, then hybridized with a digoxin-labeled DNA fragment covering the whole HBV genome.

Southern Blot Analysis of HBV DNA. Transfected cells was lysed in 750 μL NET buffer [50 mM Tris·HCl (pH 7.5), 1 mM EDTA, 100 mM NaCl, 0.5% Nonidet P-40] per 10 cm culture plate. Cell lysates were clarified by centrifugation at 12,000 × g for 30 min at 4 °C. The supernatant was adjusted to 6 mM CaCl2 and treated with 100 μg/mL of micrococcal nuclease for 30 min at 37 °C. The reaction was stopped by addition of EDTA to a final concentration of 25 mM. Proteins were digested with 0.2 mg/mL proteinase K and 0.5% SDS overnight at 37 °C. Nucleic acids were purified by phenol/chloroform extraction and ethanol precipitation. After centrifugation at 12,000 × g for 30 min at 4 °C, the pellet was resuspended in 10 μL TE buffer. DNA samples were loaded and separated by electrophoresis on a 1.2% agarose gel, transferred to a nylon membrane (Bio-Rad), and hybridized with a digoxin-labeled DNA fragment covering the entire HBx gene.

Quantitative Real-Time PCR Analysis of HBV DNA. The primers for quantitative PCR were chosen carefully from regions coding for HBV S antigen and polymerase. Sequences of primer set 1, SF1-5'-GTGTCTGCGGCGTTTTATCA-3', SR1-5'-GACAAACGGGCAACATACCTT-3'; primer set 2, PF1-5'-

TACTAGTGCCATTTGTTCAGTGG-3’, PR1-5’-CACGATGCTGTACAGACTTGG-3’. Real-time PCR analyses were performed using a SYBR Green PremixEx Taq Kit (Takara Bio, # DRR081A) in an ABI Prism 7500 PCR system (Applied Biosystems). PCR products were analyzed by fluorescence. The pHBV plasmid was used as the standard for quantification and serial dilutions from 2×10^8 to 200 IU/mL were prepared. Each standard dilution was subjected to two PCR runs in at least two independent experiments. Based on the mean threshold cycles (CT) for each dilution, a linear regression was carried out with the CT values as a function of the decadic logarithm of the number of template molecules per reaction. Least-squares regressionanalysis, performed by the ABI prism 7500, plotted CT as a function of nominal input number. The measure draw copy number from each reaction was calculated using the CT value of each PCR interpolated against the linear regression of the standard curve.
