# Supplementary material for: Structural and functional analyses of hepatitis B virus X protein BH3-like domain and Bcl-xL interaction
Source: Nat Commun. 2019 Jul 19;10:3192. doi: 10.1038/s41467-019-11173-1 (PMC6642116; doi:10.1038/s41467-019-11173-1)
Supplement: Supplementary file 3 — Reporting Summary [file 41467_2019_11173_MOESM3_ESM.pdf]

nature research

Corresponding author(s): Ning-Shao Xia, Ding Xue, Quan Yuan

Last updated by author(s): Jun 17, 2019

Reporting Summary

Nature Research wishes to improve the reproducibility of the work that we publish. This form provides structure for consistency and transparency in reporting. For further information on Nature Research policies, see Authors & Referees and the Editorial Policy Checklist .

Statistics

For all statistical analyses, confirm that the following items are present in the figure legend, table legend, main text, or Methods section.

n/a	Confirmed	
☐	☒	The exact sample size (*n*) for each experimental group/condition, given as a discrete number and unit of measurement
☐	☒	A statement on whether measurements were taken from distinct samples or whether the same sample was measured repeatedly
☐	☒	The statistical test(s) used AND whether they are one- or two-sided<br>*Only common tests should be described solely by name; describe more complex techniques in the Methods section.*
☐	☒	A description of all covariates tested
☐	☒	A description of any assumptions or corrections, such as tests of normality and adjustment for multiple comparisons
☐	☒	A full description of the statistical parameters including central tendency (e.g. means) or other basic estimates (e.g. regression coefficient) AND variation (e.g. standard deviation) or associated estimates of uncertainty (e.g. confidence intervals)
☐	☒	For null hypothesis testing, the test statistic (e.g. *F*, *t*, *r*) with confidence intervals, effect sizes, degrees of freedom and *P* value noted<br>*Give P values as exact values whenever suitable.*
☒	☐	For Bayesian analysis, information on the choice of priors and Markov chain Monte Carlo settings
☒	☐	For hierarchical and complex designs, identification of the appropriate level for tests and full reporting of outcomes
☒	☐	Estimates of effect sizes (e.g. Cohen's *d*, Pearson's *r*), indicating how they were calculated

Our web collection on statistics for biologists contains articles on many of the points above.

Software and code

Policy information about availability of computer code

Data collection	One-wavelength data set (total 180˚ with 1˚ oscillation) were collected at home X-ray source at wavelength 1.5418 Å and processed by HKL2000 (www.hkl-xray.com).
Data analysis	The structure of Bcl-xL in complex with HBx-aa113-135 was determined by Molecular replacement using the program MOLREP/CCP4 (www.ccp4.ac.uk) and coordinate from human Bcl-xL crystal structure (accession code 1R2D). The model was built by using the program O (http://xray.bmc.uu.se/alwyn) and refined using REFMAC/CCP4 (www.ccp4.ac.uk) to 2.15 Å. Statistical analyses and graphs were generated using GraphPad Prism 7.00 (GraphPad Software, La Jolla, CA, USA; www.graphpad.com).

For manuscripts utilizing custom algorithms or software that are central to the research but not yet described in published literature, software must be made available to editors/reviewers. We strongly encourage code deposition in a community repository (e.g. GitHub). See the Nature Research guidelines for submitting code & software for further information.

Data

Policy information about availability of data

All manuscripts must include a data availability statement. This statement should provide the following information, where applicable:

- Accession codes, unique identifiers, or web links for publicly available datasets
- A list of figures that have associated raw data
- A description of any restrictions on data availability

All data in this study are available from the corresponding authors upon reasonable request. The coordinate has been deposited in the Protein Data Bank, www.pdb.org, with accession number 5B1Z (Bcl-xL in complex with HBx-BH3 motif). A reporting summary for this Article is available as a Supplementary Information file. The source data for Figs 2a–b, 3b–i, 4a–d, f, h–j, 6a–g, and 7a–d and Supplementary Figs 4a–b are provided as a Source Data file.

Field-specific reporting

Please select the one below that is the best fit for your research. If you are not sure, read the appropriate sections before making your selection.

- [x] Life sciences
- [ ] Behavioural & social sciences
- [ ] Ecological, evolutionary & environmental sciences

For a reference copy of the document with all sections, see nature.com/documents/nr-reporting-summary-flat.pdf

Life sciences study design

All studies must disclose on these points even when the disclosure is negative.

Sample size	No effect size was predetermined. All the biochemical and biological experiments were performed in at least three biological replicates.
Data exclusions	No animals and/or data were excluded.
Replication	We replicated numerous data points of our study and found similar results
Randomization	Mice were randomly selected from the same pool of animals.
Blinding	All experimental procedures and quantification of results, including cell transfections, injections, tissue histological analysis, were done by two independent researchers.

Reporting for specific materials, systems and methods

We require information from authors about some types of materials, experimental systems and methods used in many studies. Here, indicate whether each material, system or method listed is relevant to your study. If you are not sure if a list item applies to your research, read the appropriate section before selecting a response.

Materials & experimental systems

n/a	Involved in the study	
[ ]	[x]	Antibodies
[ ]	[x]	Eukaryotic cell lines
[x]	[ ]	Palaeontology
[ ]	[x]	Animals and other organisms
[x]	[ ]	Human research participants
[x]	[ ]	Clinical data

Methods

n/a	Involved in the study	
[x]	[ ]	ChIP-seq
[x]	[ ]	Flow cytometry
[x]	[ ]	MRI-based neuroimaging

Antibodies

Antibodies used	The following antibodies were used in this study: anti-HBx antibody 16F9 (1:1000; developed and validated by Prof. Xia's lab). Anti-Bcl-xL antibody (1:2000; Proteintech, #a10783-1-AP). Anti-β-tublin antibody (1:2000; Abcam, #ab179513). Anti-Caspase 3 antibody (1:1000; Proteintech, #19677-1-AP). Anti-GAPDH antibody (1:5000; Proteintech, #60004-1-Ig). HRP-conjugated Goat Anti-Mouse IgG (1:5000; Proteintech, #SA00001-1). HRP-conjugated Goat-Anti-Rabbit IgG (1:5000; Thermo Fisher Scientific, #65-6120).
Validation	All the antibodies are validated by the manufacturers except for the anti-HBx 16F9, which was validated in our previous published paper (Proc Natl Acad Sci U S A. 109:18471-18476, 2012).

Eukaryotic cell lines

Policy information about cell lines

Cell line source(s)	HepG2 cell was purchased from the American Type Culture Collection (#HB-8065). HepG2.2.15 was the kind gift of Prof. Xu Lin (Fujian Medical University, China).
Authentication	Cell lines authentication was performed by short tandem repeat (STR) analysis.
Mycoplasma contamination	The cell lines have been tested for mycoplasma contamination routinely.
Commonly misidentified lines (See ICLAC register)	HepG2 is a commonly misidentified cell line, but it is a most widely used cell line in HBV research area.

Animals and other organisms

Policy information about studies involving animals; ARRIVE guidelines recommended for reporting animal research

Laboratory animals	All mice were maintained under specific pathogen-free conditions in the Laboratory Animal Centre of Xiamen University. The experiments in mice were conducted under approval of the Institutional Animal Care and Use Committee at Xiamen University and were in accordance with the Guide for the Care and Use of Laboratory Animals.<br>C57BL/6 mice (male, 6–7 week old) were divided into three groups (six mice for each injection group).
Wild animals	This study did not involve wild animals.
Field-collected samples	This study did not involve samples collected from the field.
Ethics oversight	The Institutional Animal Care and Use Committee (IACUC) at Xiamen University

Note that full information on the approval of the study protocol must also be provided in the manuscript.
